# Dorsal spine evolution in threespine sticklebacks via a splicing change in *MSX2A*

**DOI:** 10.1186/s12915-017-0456-5

**Published:** 2017-12-07

**Authors:** Timothy R. Howes, Brian R. Summers, David M. Kingsley

**Affiliations:** 1 Department of Chemical and Systems Biology, Stanford University School of Medicine, Stanford, CA USA; 2 Department of Developmental Biology, Stanford University School of Medicine, Stanford, CA USA; 3 Howard Hughes Medical Institute, Stanford University School of Medicine, Stanford, CA USA

**Keywords:** Stickleback, MSX2, msxa, Gasterosteidae, Fin spines, Acanthopterygii, Enhancers, Quantitative trait loci, Alternative splicing, Evolutionary genetics

## Abstract

**Background:**

Dorsal spine reduction in threespine sticklebacks (*Gasterosteus aculeatus*) is a classic example of recurrent skeletal evolution in nature. Sticklebacks in marine environments typically have long spines that form part of their skeletal armor. Many derived freshwater populations have evolved shorter spines. Changes in spine length are controlled in part by a quantitative trait locus (QTL) previously mapped to chromosome 4, but the causative gene and mutations underlying the repeated evolution of this interesting skeletal trait have not been identified.

**Results:**

Refined mapping of the spine length QTL shows that it lies near the *MSX2A* transcription factor gene. *MSX2A* is expressed in developing spines. In F1 marine × freshwater fish, the marine allele is preferentially expressed.

Differences in expression can be attributed to splicing regulation. Due to the use of an alternative 5 ^′^ splice site within the first exon, the freshwater allele produces greater amounts of a shortened, non-functional transcript and makes less of the full-length transcript. Sequence changes in the *MSX2A* region are shared by many freshwater fish, suggesting that repeated evolution occurs by reuse of a spine-reduction variant.

To demonstrate the effect of full-length *MSX2A* on spine length, we produced transgenic freshwater fish expressing a copy of marine *MSX2A*. The spines of the transgenic fish were significantly longer on average than those of their non-transgenic siblings, partially reversing the reduced spine lengths that have evolved in freshwater populations.

**Conclusions:**

*MSX2A* is a major gene underlying dorsal spine reduction in freshwater sticklebacks. The gene is linked to a separate gene controlling bony plate loss, helping explain the concerted effects of chromosome 4 on multiple armor-reduction traits. The nature of the molecular changes provides an interesting example of morphological evolution occurring not through a simple amino acid change, nor through a change only in gene expression levels, but through a change in the ratio of splice products encoding both normal and truncated proteins.

**Electronic supplementary material:**

The online version of this article (doi:10.1186/s12915-017-0456-5) contains supplementary material, which is available to authorized users.

## Background

The evolutionary mechanisms responsible for producing the great variety of skeletal forms in vertebrates have long been a subject of intense interest for biologists [1, 2]. As our understanding of the molecular and genetic basis of animal development has advanced, we have learned much about specific genes and pathways that control the basic formation of tissues like bone and cartilage [3, 4]. However, it remains difficult to explain how particular skeletal features evolve in wild species and to identify the individual DNA changes that underlie interesting morphological changes in nature.

Threespine sticklebacks (*Gasterosteus aculeatus*) provide an unusual opportunity to study the genetic basis of major differences in vertebrate skeletal morphology. Ocean-dwelling sticklebacks display extensive bony armor including lateral plates covering the flanks, a ventral pelvic shield, and prominent dorsal and pelvic spines. In contrast, freshwater stickleback populations have repeatedly evolved dramatic changes in the size and number of those structures, sometimes losing entire dorsal spines or the entire pelvic girdle [5]. Freshwater populations with these characteristics have arisen repeatedly over the course of 10 million years, and many of the extant populations have formed since the end of the last glacial period [6]. Despite their striking differences in appearance, the marine and freshwater populations can still interbreed, and thus they can be used in the laboratory to generate large crosses between divergent parents for the mapping of quantitative trait loci (QTL). This can lead to the identification of specific chromosomal regions and specific genes that contribute to interesting evolutionary differences [7].

Stickleback genes affecting lateral plates, pelvic spines, pharyngeal teeth, and ventral pigmentation have been identified by QTL mapping [8–13]. Perhaps the most conspicuous feature of sticklebacks, however, is the one for which they are named: the dorsal spines. Although developmentally related to the skeletal rays that support a variety of movable soft fin surfaces in teleosts [14], the dorsal spines of sticklebacks are rigid freestanding structures that articulate in a lockable hinge joint with underlying skeletal structures and can be raised and lowered in mating displays or as a defense against soft-mouthed predators [15]. Dorsal spines show great variation between stickleback populations, with many freshwater fish showing spine reduction, sometimes including loss of one or more spines [16–21]. Spine number varies even more greatly across different stickleback species, which bear names such as fourspine, ninespine, and fifteenspine stickleback [22]. Clearly, spines are a skeletal feature that can undergo dramatic changes as sticklebacks evolve in different environments, and the repeated nature of spine modifications suggests that these changes are adaptive.

Previous genetic mapping studies have identified broad chromosome regions contributing to spine length differences between stickleback populations [23–25]. In a cross between long-spined marine fish and extremely spine-reduced Paxton Lake benthic sticklebacks, the locus with the largest effect on dorsal spine length maps to chromosome 4 [25], the same chromosome that harbors the ectodysplasin (*EDA*) locus, which controls major changes in armor plate number in sticklebacks [8]. Here, we report additional fine mapping of the dorsal spine QTL region on chromosome 4, and we identify the homeodomain transcription factor gene *MSX2A* as a major contributor to dorsal spine variation between marine and freshwater sticklebacks. The expression pattern, molecular changes, and ability of *MSX2A* to alter spine length provide a new example of the molecular basis of repeated skeletal evolution in vertebrates.

## Results

### Dorsal spine and anal spine lengths map to chromosome 4

**Fig. 1.**
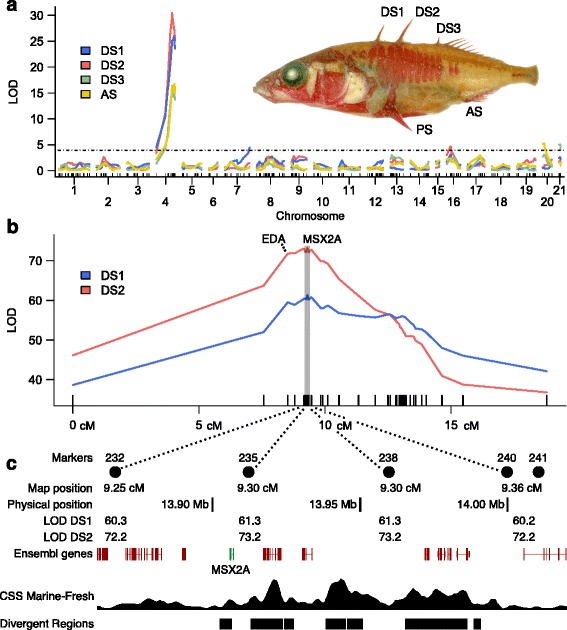
High-resolution mapping of a major QTL controlling dorsal spine length in sticklebacks. **a** QTL scan results for spine lengths in a family of 375 F2s from the JAMA × PAXB cross. Positions of dorsal spines 1–3, the anal spine, and pelvic spines (DS1–DS3, AS, and PS) are indicated on an alizarin-stained stickleback. The dashed line shows an *α*=0.05 permutation-based threshold. **b** Fine mapping for a subset of chromosome 4 containing the peak from (**a**). DS1 and DS2 were measured in additional recombinant fish from the F2 cross and genotyped more densely to refine the QTL position. Marker positions are indicated by tick marks along the bottom. The highlighted area around the peak indicates the region displayed in (**c**). **c** Genome browser view of the physical region surrounding the QTL peak. The peak marker, MEM235, is located at 13,912,800 bp (between genes *MSX2A* and *CPEB4A*). The marker MEM238 has an identical position on the linkage map, with a physical position of 13,965,800 bp (between *STC2A* and *NKX2-5*). The browser tracks show the marine/freshwater cluster separation score (CSS) and divergent regions identified in Jones et al. [27]. These represent regions showing consistent sequence differences between marine and freshwater populations, as identified by the analysis of 21 genomes using CSS and a self-organizing map/hidden Markov model method. Ensembl gene models are shown in red, with *MSX2A* highlighted in green. From left to right, the genes shown are *RNF44*, *FAF2*, *PIN4*, *TSSK1*, *DRD1A*, *MSX2A*, *CPEB4A*, *STC2A*, *NKX2-5*, *BNIP1A*, *ATP6V0E1*, *RPL26*, and *PPP2R2BA*. Names appended with *A* are duplicated with respect to the human genome and have a corresponding *B* paralog on chromosome 7. AS anal spine, CSS cluster separation score, DS dorsal spine, EDA ectodysplasin, JAMA Japanese marine population, LOD logarithm (base 10) of odds ratio for QTL model at a given locus, PAXB Paxton Lake benthic population, PS pelvic spine

A large F2 cross derived from a Japanese marine stickleback (JAMA) and a freshwater benthic stickleback from Paxton Lake, British Columbia (PAXB), has previously been used to map QTL for lateral plate number, pelvic spine length, ventral pigmentation, and many components of the axial and branchial skeleton [9, 10, 25, 26]. Using a set of 375 F2 fish from a single pair of F1 parents, we found that the lengths of the three dorsal spines and anal spine are all influenced by a major QTL on chromosome 4 (Fig. 1 a). The LOD scores (logarithm of odds ratio for a QTL) range from 17.9 to 36.0, with percentage variance explained (PVE) of 18.1 to 31.6 % (Table 1).

**Table 1:** QTL identified for DS1, DS2, DS3, and AS in Family 4

						Phenotype means			
Trait	Chromosome	Position (cM)	Marker	LOD	PVE	M_1_M_2_	F_1_M_2_	M_1_F_2_	F_1_F_2_
DS1	4	61.4	Stn47	27.03	26.47	0.39	0.06	−0.13	−0.31
DS1	7	85.8	Stn82	4.60	3.88	0.08	0.13	−0.04	−0.17
DS1	16	13.5	Stn175	4.45	3.75	0.14	−0.03	0.02	−0.13
DS1		Full model		34.80	35.93				
DS2	4	55.3	Stn45	35.95	31.56	0.39	0.05	−0.19	−0.24
DS2	8	42.0	Stn87	3.96	2.83	0.03	0.11	−0.04	−0.12
DS2	9	8.7	Stn108	5.69	4.11	0.13	−0.07	−0.06	−0.04
DS2	16	13.5	Stn175	7.38	5.38	0.18	−0.01	−0.06	−0.09
DS2		Full model		46.41	43.71				
DS3	4	55.0	Gac4174	17.93	18.06	0.12	0.01	−0.04	−0.08
DS3	13	4.8	Stn149	5.04	4.68	−0.02	0.04	−0.07	0.03
DS3	21	0.0	Stn218	6.45	6.04	0.05	0.01	0.01	−0.07
DS3		Full model		26.48	28.20				
AS	4	63.5	Stn292	19.11	20.81	0.14	−0.00	−0.04	−0.08
AS	17	45.8	Stn273	4.40	4.31	−0.06	0.02	−0.01	0.03
AS	20	0.0	Stn213	5.66	5.60	0.06	−0.02	0.01	−0.07
AS		Full model		26.87	30.96				

The stepwise model selection method resulted in a multi-QTL additive model for each trait. The rows describing individual QTL contain position information, mean phenotypes for each genotype class at the QTL position, and LOD and PVE values that compare the full model to a sub-model with the individual QTL term dropped. Rows labeled “Full model” show the LOD and PVE scores for the additive model that includes all of the QTL terms. Genotype classes are labeled according to the grandparent of origin of each allele. Phenotype values are residuals to standard length in millimeters

*AS* anal spine, *DS* dorsal spine, *F* freshwater, *LOD* logarithm (base 10) of odds ratio for QTL model, *M* marine, *PVE* percentage variance explained, *QTL* quantitative trait locus

To determine the position of this QTL more precisely, we used additional fish from other families in the cross (derived from the same JAMA and PAXB grandparents, but different sets of F1 parents). F2s from six additional families were genotyped using six markers on chromosome 4 (Stn42, Stn45, Stn183, Stn266, Stn292, and Stn309), and fish that were recombinant within that interval were further genotyped with a total of 48 densely spaced markers (Additional file 1: Table S1). Dorsal spines 1 and 2 (DS1 and DS2) were measured on recombinant individuals, and QTL analysis of chromosome 4 identified peak LOD scores at marker MEM235 for both DS1 and DS2 (Fig. 1 b). The LOD and PVE values for DS1 and DS2, respectively, were 66.3 (28.4 % PVE) and 73.2 (30.2 % PVE; Table 2). The adjacent markers on either side of the peak have scores approximately 1 LOD less than the peak, but the scores rise again for more distant markers, creating a disjoint 1-LOD interval that covers 1 cM on the linkage map (MEM006 to MEM253). For DS1, there was evidence for an additive model combining two separate QTLs on chromosome 4 (Table 2), the main peak located at 9.3 cM (MEM235) and a secondary peak at 13.2 cM (BRS18).

**Table 2:** QTL identified by fine mapping on chromosome 4

					Phenotype means		
Trait	Position (cM)	Marker	LOD	PVE	MM	MF	FF
DS1	9.3	MEM235	10.69	3.97	0.38	−0.04	−0.32
DS1	13.2	BRS18	4.93	1.81	0.35	−0.01	−0.34
DS1	Full model		66.28	28.44			
DS2	9.3	MEM235	73.16	30.23	0.38	−0.05	−0.29

LOD scores, percentage variance explained (PVE), and mean trait values for each genotype class at the marker position are shown. For DS1, an additive model was identified that combines two QTLs located at 9.3 and 13.2cM. The first two rows indicate the LOD and PVE associated with dropping one QTL from the model. The third row shows the LOD and PVE for the full model combining the two QTLs. Genotype classes are labeled according to grandparent of origin. Phenotype values are residuals to standard length in millimeters

*DS* dorsal spine, *F* freshwater, *LOD* logarithm (base 10) of odds ratio for QTL model, *M* marine, *PVE* percentage variance explained, *QTL* quantitative trait locus

For the QTL mapping, we excluded markers that had the same genetic position as other markers. The marker MEM238 had the same genotype results and map position as MEM235, so there is equal evidence for the peak being located at MEM235 (13,912,000 bp, between *MSX2A* and *CPEB4A*) or MEM238 (13,965,800 bp, between *STC2A* and *NKX2-5*). Physical positions refer to the BROADS1/gasAcu1 assembly [27].

### Candidate gene *MSX2A* shows allele-specific differences in transcript levels in F1 hybrids

The gene *MSX2A* lies adjacent to peak marker MEM235, and it stands out as a likely candidate for a gene affecting a skeletal trait. It encodes a homeodomain transcription factor that has known roles in osteoblast differentiation and associations with skeletal phenotypes [28, 29]. In zebrafish, multiple *msx* paralogs are expressed during regeneration of the fin rays, which are developmentally related to dorsal spines [30]. Based on the QTL mapping data and the known associations with skeletal traits in other organisms, we tested whether *MSX2A* showed relevant changes in its sequence or expression pattern that could explain the divergent phenotypes of marine and freshwater sticklebacks.

**Fig. 2.**
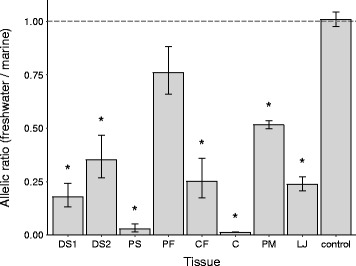
Allele-specific expression of *MSX2A* in F1 hybrid fish. Expression ratios of freshwater (PAXB) to marine (RABS) transcript, as measured by pyrosequencing of an intron-spanning PCR product in F1 hybrids. DS1, DS2, PS, CF, C, PM, and LJ showed significant differences (*) from a 50:50 control plasmid mixture. Error bars represent standard error of the mean (SEM). C cloaca, CF caudal fin, DS dorsal spine, LJ lower jaw, PAXB Paxton Lake benthic population, PF pectoral fin, PM premaxilla, PS pelvic spine, RABS Rabbit Slough marine population

Previous studies suggest that regulatory alterations underlie more than 80 % of the adaptive loci contributing to repeated stickleback evolution [27]. To test for possible *cis*-regulatory differences between marine and freshwater sticklebacks at the *MSX2A* locus, we generated F1 hybrid fish, which have both alleles expressed within the same *trans*-acting environment. The relative amounts of marine and freshwater transcripts were quantified by pyrosequencing [31] using intron-spanning primers. In multiple tissues, there were markedly fewer copies of the freshwater allele relative to the marine allele, including in DS1 and DS2, the pelvic spines, and the oral jaws (Fig. 2). In contrast, a different gene in the QTL interval, *CPEB4A*, showed balanced expression of the marine and freshwater alleles in the F1 hybrids (Additional file 2: Figure S1). These data indicate that there are significant *cis*-acting differences that affect *MSX2A* transcript levels in marine and freshwater fish. We note, however, that the primers used for pyrosequencing did not detect alternative splice forms. Subsequent results suggest that reduced levels of the full-length transcript in freshwater fish likely arise from *cis*-acting splicing differences that increase the production of an alternative, shorter transcript (see below).

### A conserved non-coding region drives *MSX2A* expression in developing skeletal structures

**Fig. 3.**
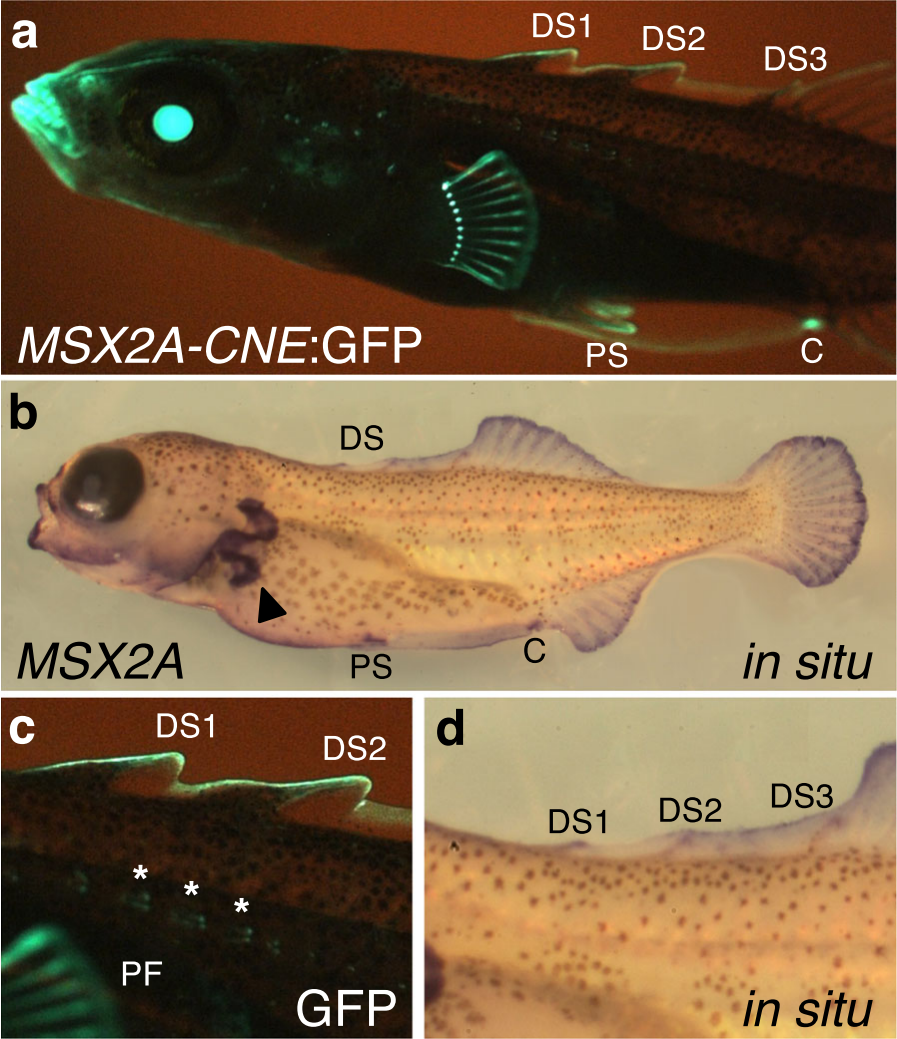
A non-coding enhancer sequence recapitulates endogenous *MSX2A* expression patterns in developing spines and fins. **a** GFP expression in a 26-day-old transgenic stickleback larva driven by the stickleback *MSX2A-CNE* enhancer and zebrafish *hsp70* promoter. Structures showing expression include dorsal spines, pectoral and median fin rays, cloaca, anal spine, pelvic spines, median fin fold, jaws, teeth, nostrils, eye, and lateral line neuromasts. Eye (lens) expression is a regular background pattern of the *hsp70* promoter. The image is horizontally reversed from the original. **b** RNA in situ hybridization in a 20-day-old marine stickleback larva, showing labeling in presumptive dorsal spines forming within the fin fold, as well as pelvis, cloaca, nostrils, gills, median fins, and pectoral fins. **c**, **d** Closer view of dorsal spines in the GFP and in situ larvae. In both the GFP transgenics and the in situ larvae, expression in spines is stronger near the distal ends. GFP fluorescence is strong in the fin fold surrounding the dorsal spines, with lighter signal in the spines themselves. In the GFP panel, the pectoral fin and three of the neuromasts (*) are also indicated. C cloaca, DS dorsal spines, GFP green fluorescent protein, PF pectoral fin, PS pelvic spine

To identify possible regulatory sequences controlling *MSX2A* expression, we cloned conserved non-coding elements (CNEs) found near the gene and tested them for enhancer activity in transgenic sticklebacks using a green fluorescent protein (GFP) reporter vector with an *hsp70* minimal promoter (Additional file 2: Figure S2). This vector was injected into single-cell embryos, and the results were assessed in transient transgenic fish. A 664-bp CNE near the start of the gene (*MSX2A-CNE*; Additional file 2: Figure S3) was found to produce a complex expression pattern in developing larvae, including prominent expression in the dorsal spines, as well as the anal spine and pelvic spines, and all of the fin rays of the median fins and paired fins (Fig. 3 a). Other sites of expression include the jaws, cloaca, and neuromasts of the lateral line. In embryonic stages, GFP expression is observed in the developing fin folds and sensory placodes (Additional file 2: Figure S4). Expression in the eyes at all stages can be attributed to the zebrafish *hsp70* promoter used in the expression construct, which has known background activity in the lens [32].

The GFP expression pattern largely recapitulates the pattern observed by RNA in situ hybridization in developing stickleback larvae (Fig. 3 b). The early embryonic pattern also matches reported in situ data for zebrafish [30, 33, 34]. In the stickleback larval in situ experiments, staining is observed in the median fin fold, particularly at the sites of the developing dorsal spines (Fig. 3 d). Staining of RNA transcripts is also observed in the jaws, pectoral fins, pelvic spines, and cloaca.

There are a small number of sequence differences between the marine and freshwater alleles of the *MSX2A-CNE*. However, the marine and freshwater alleles produce very similar GFP expression patterns in transgenic sticklebacks (data not shown). Thus, changes outside the CNE are likely to be responsible for the *cis*-acting differences seen in the allele-specific expression experiments.

### The freshwater allele produces a shorter *MSX2A* transcript

**Fig. 4.**
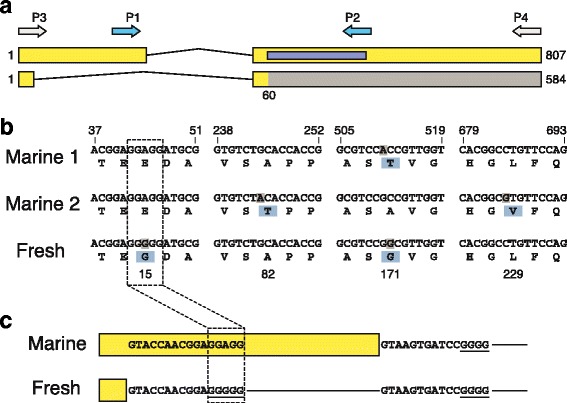
Structure and alternative splicing of stickleback *MSX2A*. **a** Transcript diagram of *MSX2A*, showing exons (yellow segments) and intron (connecting line). Both a full-length transcript (above) and a shortened transcript based on an alternative 5 ^′^ splice site can be observed by RT-PCR. Primer binding sites used for the allele-specific expression assay (P1 and P2) and for RT-PCR (P3 and P4) are shown. The region encoding the homeodomain (DNA-binding domain) is highlighted in purple. The intron spans approximately 580 bp (800 bp if the alternative splice site is used) and is not shown to scale. In the shortened transcript, a stop codon occurs 30 bp into the second exon, and the remainder of the exon is untranslated (shown in gray). **b** Selected parts of a sequence alignment between the freshwater allele and two versions of the marine allele, highlighting sites where nucleotide changes lead to amino acid changes. Nucleotide coordinates are listed above; amino acid positions are indicated below. The only consistent amino acid difference between the freshwater allele and both marine alleles is the E15G polymorphism. The nucleotide change at that site also creates a five-nucleotide poly-G tract (GGGGG) that is specific to the freshwater allele. **c** The freshwater poly-G tract is a motif preferred by hnRNP F/H family proteins, the binding of which can promote the usage of an adjacent 5 ^′^ splice site [45]. Self-interaction between hnRNP proteins bound at two different sites can also work to define the intervening sequence as intronic [46]. The presence of poly-G tracts near both the normal 5 ^′^ splice site and the alternative site in the freshwater allele (underlined) suggests that an hnRNP interaction could occur and promote usage of the alternative site. hnRNP heterogeneous nuclear ribonucleoprotein, RT-PCR reverse-transcription polymerase chain reaction

While cloning the *MSX2A* coding sequence from cDNA, we discovered that an alternatives, shorter transcript can occur. The expected full-length transcript is 807 bp, but the short transcript is 584 bp. Cloning and sequencing of the short product revealed that it is missing most of the first exon, due to an alternative 5 ^′^ splice site located within the first exon (Fig. 4 a). When the alternative splice donor is used, only the first 30 bp of exon 1 is retained in the resulting transcript. As a result, there is a frameshift that affects exon 2 and creates an early stop codon. The peptide encoded by this transcript would be only 19 residues long, and would lack the DNA-binding domain normally found in exon 2.

To compare the relative levels of full-length and short transcripts produced by the marine and freshwater alleles, we used reverse-transcription polymerase chain reaction (RT-PCR) to amplify both *MSX2A* isoforms from F1 marine/freshwater hybrid tissue samples (Little Campbell River marine population (British Columbia) × PAXB; Additional file 2: Figure S5a). We then purified the upper and lower bands from an electrophoresis gel, and cloned and sequenced individual amplicons from each band to determine whether they originated from the marine or freshwater chromosome. Out of 44 clones obtained from the upper band, 43 came from the marine allele, and only one came from the freshwater allele. Excluding clones of off-target amplicons, 14 out of 15 clones derived from the lower band came from the freshwater *MSX2A* allele, and one came from the marine allele. The differential recovery of marine and freshwater alleles in the two PCR products was significant, considering a null hypothesis of equal likelihood of recovery (*p*=5×10^−12^ and *p*=0.001, respectively; binomial test).

To test further for allelic bias in the two different isoforms, we again purified the upper and lower bands from an RT-PCR reaction. The purified bands were incubated in a restriction digest with the enzyme *BspCNI*, which cuts the freshwater allele at three sites but cuts the marine allele only twice. The migration patterns of the digest products were consistent with a largely marine origin for the upper band and a freshwater origin for the lower band (Additional file 2: Figure S5b). The marine origin of the upper band is consistent with the pyrosequencing-based allele-specific expression results, which showed greater expression of the full-length transcript from the marine allele.

### An A-to-G change produces a novel poly-G splicing enhancer motif in freshwater fish

To determine whether sequence differences between the marine and freshwater *MSX2A* alleles might influence usage of the alternative splice donor, we compared the sequences of the three main alleles identified by sequencing exons 1 and 2 in four long-spined and ten spine-reduced populations. There are three single-nucleotide differences that distinguish the allele shared by PAXB and other freshwater populations from the alleles we commonly found in marine fish (two of the changes are non-synonymous and shown in Fig. 4 b; full exon sequences from each population are shown in Additional file 3). None of these sequence differences has a direct effect on the alternative splice donor site. However, an A-to-G nucleotide change in freshwater fish at the +44 position causes a predicted amino acid substitution (Glu to Gly), and also generates a novel poly-G motif 12 nt downstream of the alternative splice site in exon 1 (Fig. 4 c). Previous studies have shown that GGGGG sequences can serve as splicing enhancers, and the change from a GGAGG to a GGGGG sequence in the freshwater *MSX2A* gene may, therefore, underlie the preference for the alternative splice site (see “Discussion”).

### Expression of the marine *MSX2A* allele affects dorsal spine length in transgenic freshwater sticklebacks

**Fig. 5.**
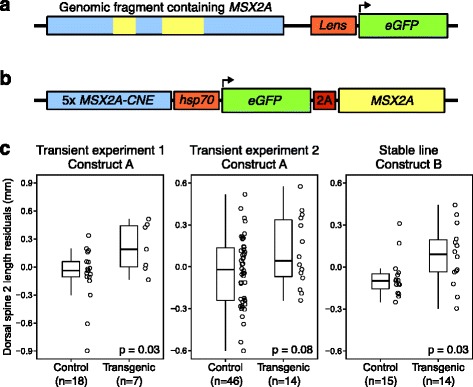
Phenotype rescue by transgenic *MSX2A* expression. **a** Construct A contains a 5.6 kb genomic fragment that includes the two exons of *MSX2A* (yellow) and surrounding non-coding DNA (blue). An *eGFP* reporter with lens-specific promoter is included to help identify transgenics. **b** Construct B expresses both *eGFP* and *MSX2A* under the control of the *MSX2A-CNE* (cloned as five concatenated copies) and an inducible *hsp70* promoter. The 2A peptide linker allows expression of two products from a single transcript. **c** Results from three experiments in which the marine *MSX2A* allele was expressed in transgenic freshwater fish (PAXB population). The first two experiments compare GFP-expressing mosaic transgenics injected with construct A to sibling controls that either were not injected or showed no expression. The third experiment results from the cross of a construct B founder transgenic fish to a wild-type PAXB fish. GFP-expressing fish (carriers of the stable transgene) were compared to siblings lacking the transgene. Length measurements are the residuals of DS2 length from a linear regression on standard length. *P*-values are from Welch’s *t*-test. eGFP enhanced green fluorescent protein, PAXB Paxton Lake benthic population

To determine whether the expression level of the full-length *MSX2A* transcript has an effect on stickleback skeletal traits, we injected the cloned coding sequence of a full-length marine *MSX2A* allele into the embryos of freshwater PAXB fish. One expression construct (construct A) was built with a 5.6 kb genomic fragment containing the marine *MSX2A* gene, including its intron and surrounding non-coding DNA such as the *MSX2A-CNE* (Fig. 5 a; Additional file 2: Figure S3). The vector backbone also included *enhanced green fluorescent protein* (*eGFP*) with a lens-specific promoter (gamma F-crystallin promoter) for easy identification of transgenic animals. Two transient transgenesis experiments were conducted, and injected embryos showing green fluorescence in the lens were compared to siblings that were not injected or were injected but not fluorescent (Fig. 5 c). Linear regression of dorsal spine length on standard length was used to calculate residuals of DS2 length for each fish (see Additional file 4 for details of calculations and comparisons). DS1 and pelvic spines were absent in most of the fish from the PAXB genetic background and were not considered (the absence of these structures limits analysis to DS2, but makes it possible to carry out the experiments in the same spine-reduced population originally used for QTL mapping). In the first experiment, a significant difference between groups was observed (*t*(12.2)=2.40, *p*=0.03, Welch’s *t*-test), with transgenic fish having average DS2 residuals 0.29 mm longer than controls. The second experiment also had average DS2 residuals in transgenics that were longer than controls, by 0.14 mm, though the difference did not show statistical significance at a threshold of *α*=0.05 (*t*(21.1)=1.84, *p*=0.08, Welch’s *t*-test). The latter experiment used more fish but grew them more densely in tanks, and there were tank-to-tank differences in body size. The tanks of uninjected fish had greater attrition, and the resulting lower density caused them to grow larger, an effect that may not be fully corrected by taking residuals. See “Outlier analysis” in “Methods” for additional information.

We generated a second expression construct (construct B) that used the *MSX2A-CNE* to provide an appropriate expression pattern. The *MSX2A-CNE* was cloned upstream of the full-length marine *MSX2A* open reading frame, which was linked to *eGFP* by a 2A peptide sequence [35] to allow simultaneous expression of both the MSX2A gene product and a GFP reporter (Fig. 5 b). Embryos injected with this construct showed high mosaicism, but we were able to cross a GFP-expressing transgenic founder male with a wild-type PAXB female to produce a stable transgenic line (Fig. 5 c, last panel). The stable transgenic progeny displayed non-mosaic GFP expression; these individuals (*n*=14) were raised to maturity and compared to a set of non-transgenic sibling controls (*n*=15). The transgenic fish had longer dorsal spines after correcting for standard length (0.16 mm longer on average; *t*(22.6)=2.26, *p*=0.03, Welch’s *t*-test). Thus, in an experiment using a stable, non-mosaic transgene, marine *MSX2A* was found to significantly lengthen the characteristically reduced dorsal spines that have evolved in freshwater sticklebacks.

## Discussion

### Role of *MSX2A* in stickleback skeletal evolution

We have shown that stickleback dorsal spine length, like lateral plate number, is controlled by a major QTL on chromosome 4. Although *EDA* is known to control median fin structures in other fish [34], our population studies and fine-mapping experiments indicate that the major dorsal spine QTL in sticklebacks is distinct from the major lateral plate QTL at *EDA*. The spine length QTL resolves to a location approximately 1 megabase away from the *EDA* locus, near the transcription factor gene *MSX2A* (Fig. 1). The expression pattern of *MSX2A* in sticklebacks is consistent with a key role in spine development (Fig. 3). The gene shows significant marine/freshwater differences in allele-specific expression experiments (Fig. 2), and expression of a stable *MSX2A* transgene significantly increases the size of freshwater spines (Fig. 5). *MSX2A* thus provides a new example of a major developmental control gene that contributes to skeletal evolution in natural populations.

Although our transgenic PAXB sticklebacks expressing full-length marine *MSX2A* showed increases in the lengths of their dorsal spines (0.14–0.29 mm, statistically significant in two of the three experiments), the lengths obtained did not approach the lengths observed in marine fish. However, the differences between the transgenic and non-transgenic groups were similar to the difference between the marine/freshwater heterozygote and freshwater homozygote genotype groups in the F2 cross (0.24 mm; Table 2). Full restoration of the marine phenotype would likely require additional contributions from different genes, as the *MSX2A* locus accounted for only 20–30 % of the overall spine length variance in our cross. It is also possible that there are multiple linked loci on chromosome 4 that affect the trait, with the 20–30 % variance explained representing the combined effects of *MSX2A* and other linked genes such as *STC2A* and/or *EDA*. In general, many morphological traits map to chromosome 4, and our fine-mapping results for spine length support the presence of at least one additional QTL affecting DS1 (Fig. 1 b; causative gene unknown).

In a comparison of many marine and freshwater stickleback genomes [27], the *MSX2A* locus shows strong signs of repeated sequence differentiation through reuse of standing variation, much like the *EDA* locus [8] (Fig. 1 c). This suggests that *MSX2A* plays a similar role in facilitating the parallel evolution of armor changes in freshwater habitats. Selection pressures in many freshwater habitats evidently favor reduction of the spiny armor [16]. Reimchen [19] has suggested that spine reduction is adaptive in some freshwater habitats because heavily armored sticklebacks face greater predation from insect predators that grasp their prey, an idea that is supported by temporal variation in spine numbers [36] and by selection experiments [37]. In contrast, retention of robust spines should be favored by selection in ocean and lake environments where there are large predatory fish [15, 16, 38].

Close genetic and physical linkage between the major locus that controls armor plate patterning in sticklebacks (*EDA*) and a separate major locus controlling the length of the dorsal spines (*MSX2A*) would facilitate co-inheritance of either high armor and long spines or low armor and short spines following hybridization between marine and freshwater fish. These matched pairs of traits could then favor survival in contrasting environmental conditions. Clustering of QTLs for ecologically relevant traits has been predicted to evolve in species where contrasting ecotypes can still meet and hybridize [39, 40]. Although clustered QTLs have frequently been observed in stickleback genetic crosses, it has been difficult to determine whether the apparent clustering is due to the pleiotropic effects of a single locus or to multiple linked loci controlling different traits [25, 41]. Our results provide a clear example of two different armor traits that are controlled by two separate but linked developmental genes, with the linkage between them causing armor plate reduction and spine reduction to be co-inherited in crosses between divergent sticklebacks. These results contrast with recent studies of other classic morphological trait clusters, such as wing color patterns in *Heliconius*, where multiple different color elements are controlled by changes in different enhancers of a single developmental control gene [42, 43]. *EDA* and *MSX2A* are also physically linked in the genomes of other fish species, such as Nile tilapia, which has dorsal spines but does not develop lateral armor plates. The linkage between armor and spine traits in sticklebacks is thus based on ancient synteny, rather than arising from a novel rearrangement of genes within the stickleback group.

### Allele-specific expression vs allele-specific splicing differences

Previous studies of the genes underlying recurrent stickleback evolution have found that *cis*-regulatory changes play an important role [8–10, 27]. Repeated examples of pelvic spine loss, for example, are caused by loss of an enhancer sequence near *PITX1* that guides expression in the developing pelvic fin buds [11]. Although the tissue-specific pelvic enhancer is lost, the *PITX1* gene is left intact and continues to be expressed normally in the jaws and brain. Similarly, changes in the lateral plate number are associated with a single base-pair change in a tissue-specific enhancer affecting *EDA* [44]. A loss-of-function allele in the *EDA* coding sequence would have major side effects on structures such as fins and teeth [34], but the regulatory mutation that occurred in sticklebacks has a targeted effect on the posterior lateral plates.

We tested for allele-specific expression of *MSX2A* in F1 hybrids to determine whether *MSX2A* is also subject to a tissue-specific regulatory change in freshwater populations. We did find reduced levels of a particular freshwater transcript in several tissues, including dorsal spines, pelvic spines, and other sites of *MSX2A* expression (Fig. 2). We also identified a conserved enhancer sequence (*MSX2A-CNE*) that drives expression in the developing spines (Fig. 3). There are a small number of sequence differences between the marine and freshwater versions of the enhancer, but tests of the two versions in transgenic fish did not produce obvious differences in expression patterns. GFP expression in transient transgenic animals exhibits variation due to differences in transgene integration sites and mosaicism within each embryo, so we cannot exclude the possibility of a subtle quantitative effect on expression levels due to sequence changes in this enhancer or due to changes in other enhancers that remain to be characterized.

An alternative mechanistic explanation emerged when we found that there are multiple splice forms of the *MSX2A* gene in sticklebacks (Fig. 4). The alternative splice form is shorter and lacks the functional protein-coding domains of the gene, so we can expect loss of *MSX2A* function to the extent that the short splice product predominates over the full-length form. In F1 hybrid animals, we indeed found that the short product comes largely from the freshwater allele and the full-length form comes predominantly from the marine allele (Additional file 2: Figure S5). Thus, there is an allele-specific difference in the splicing patterns of the stickleback *MSX2A* gene. The pyrosequencing-based allele-specific expression results are consistent with this interpretation (Fig. 2). The primers used in that experiment are specific to the full-length transcript, so the apparent reduction of *MSX2A* expression from the freshwater allele likely reflects a greater bias toward production of the short splice product.

### Role of the sequence change near the alternative splice site

Our sequence analysis indicates that the freshwater *MSX2A* allele has acquired a novel splicing enhancer motif through a single-nucleotide change. The sequence GGAGG in marine fish has changed to GGGGG in freshwater fish, creating a poly-G tract near the alternative splice site. Previous studies have shown that poly-G tracts of at least 3 nt can act as binding sites for spliceosome proteins such as heterogeneous nuclear ribonucleoprotein (hnRNP) A/B and hnRNP F/H, with 4 or 5 nt being optimal for certain hnRNPs [45]. Binding of these hnRNPs to poly-G tracts can promote selection of nearby 5 ^′^ splice sites. In particular, when there are two alternative sites, each with a poly-G motif, the more distal 5 ^′^ splice site is favored due to self-interactions of the hnRNPs and looping out of the intervening sequence [45, 46]. This is, in fact, the case for freshwater *MSX2A*, as the normal 5 ^′^ splice site has its own nearby GGGG motif, which can partner with the GGGGG motif near the alternative site (Fig. 4 c).

Another factor affecting splice site selection near poly-G motifs is the strength of the splice site, as defined by Xiao et al. [47]. The alternative 5 ^′^ splice site within the first *MSX2A* exon is classified as an intermediate-strength site, which is the type that is expected to be strongly affected by the activity of a nearby poly-G motif.

Investigations of the evolution of development have often focused on the relative contributions of protein-coding changes and regulatory sequence changes [48]. Protein-coding changes can have dramatic phenotypic effects, but they typically affect the function of a gene in all of the tissues where it is expressed, whereas regulatory changes can target specific tissues. In the case of *MSX2A* and dorsal spine evolution in sticklebacks, we have encountered an interesting mechanism that greatly affects the protein product but occurs through regulatory change. A base-pair change within the coding sequence creates a new regulatory element that produces a non-functional protein by favoring an alternative splice site. However, the resulting loss of function is likely incomplete due to lingering production of some full-length transcript. Furthermore, the splicing event could be subject to tissue-specific regulation, particularly if the mRNA-binding factors that guide splice site selection are themselves expressed in a tissue-specific manner [49]. The different allelic ratios observed in the pyrosequencing experiment show that full-length *MSX2A* expression does vary between tissues (e.g., a strong marine bias in spines and no significant difference in the pectoral fin; Fig. 2).

### Loss of *MSX2A* in acanthopterygians lacking bony armor

**Fig. 6.**
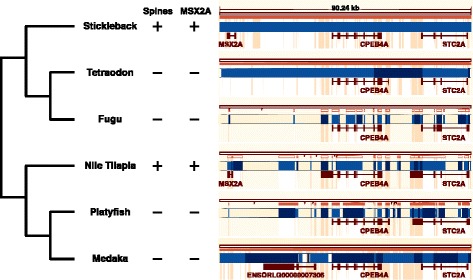
Alignment of the *MSX2A*–*CPEB4A*–*STC2A* region in acanthopterygian genomes. Ensembl 82 genome browser view [65] of an alignment between the stickleback genome and the genomes of tetraodon, fugu, tilapia, platyfish, and medaka. These species share a common ancestor that had spiny fin rays [66, 67], and spines have been independently lost in the pufferfish (tetraodon and fugu) and in medaka and platyfish. Blue boxes are scaffolds and contigs, and red boxes are exons of Ensembl gene models. Orange highlighting indicates regions of sequence alignment with the stickleback genome. Narrow orange strips indicate whether the alignment is on the same strand (solid) or the opposite strand (hollow) relative to the stickleback genome. Black tick marks indicate changes between chromosomes (or scaffolds in incompletely assembled regions). In the *MSX2A* region, partial alignments in fugu and platyfish correspond to *msx* paralogs on other chromosomes. Partial alignment in the medaka genome corresponds to an *MSX2A* pseudogene

Many species outside the stickleback group show variation in their dorsal spines. Acanthopterygii is a superorder of fish characterized by the presence of stiff spiny rays at the anterior of their dorsal and anal fins [50]. This group encompasses sticklebacks along with thousands of species representing most of the morphological diversity of the ray-finned fishes. Although the spines are thought to serve a protective function in many species, there have been independent losses of spines in different sub-groups, such as pufferfish (Tetraodontiformes) and the clade containing medaka and platyfish (Beloniformes + Cyprinodontiformes). The available genome sequences of several species that have lost their bony spines (two puffers, medaka, and platyfish), in comparison to species that still retain their bony spines (stickleback and tilapia), suggest that at least two phylogenetically independent examples of spine loss have been accompanied by independent losses of the *MSX2A* gene (Fig. 6). This raises the possibility that changes at the *MSX2A* locus could be related to loss of spiny armor outside of sticklebacks, either in a causative manner (loss of the gene leading to loss of spines) or through neutral loss of the gene following independent loss of the skeletal structures where *MSX2A* is normally expressed. It may be possible to test these hypotheses using structural and functional approaches like those we have used for sticklebacks, including comparison of gene structures in closely related species that differ in the presence or absence of spines, and reintroduction of the *MSX2A* gene by transgenic approaches to test for phenotypic effects on evolved skeletal structures. Although targeted gene replacements have traditionally not been possible outside a limited range of model organisms, methods for precise gene editing are rapidly advancing in sticklebacks and other species [51, 52]. It may soon be possible to recreate the characteristic poly-G sequence of the freshwater *MSX2A* gene in a marine background, or generate other *MSX2A* mutations in sticklebacks or other species, to test more precisely the effects of specific DNA base-pair changes on both MSX2A function and skeletal morphology.

## Conclusions

Major components of the skeletal armor of freshwater sticklebacks have been reduced through changes in key developmental genes. For lateral plates, pelvic spines, and now dorsal spines, we can identify *EDA*, *PITX1*, and *MSX2A*, respectively, as major loci controlling armor reduction. *MSX2A* and *EDA* are physically linked on stickleback chromosome 4, helping explain previous observations that spine reduction and armor plate reduction often co-vary in crosses. A genetic cluster or supergene of morphological traits on stickleback chromosome 4 has thus arisen in part through changes in separate, linked genes, and not purely through the pleiotropic effects of a particular mutation or through multiple changes in the regulatory elements of a single master control gene.

For *EDA* and *PITX1*, the causal mutations in freshwater populations have previously been traced to alterations in tissue-specific transcriptional enhancers. Mutations in such enhancers provide a commonly recognized mechanism for preserving the essential functions of a key developmental gene, while avoiding the multiple negative pleiotropic effects that might arise from disrupting the coding sequence and changing the structure of the gene’s product. Our studies of *MSX2A* provide an interesting alternative mechanism for avoiding negative pleiotropy. We have identified a specific base-pair change in freshwater fish that generates a new splicing enhancer sequence in the *MSX2A* gene. Although increased use of an alternative splice donor site in freshwater fish leads to an increased number of transcripts encoding a severely truncated *MSX2A* protein, the change is quantitative, and full-length transcripts are also still produced that code for normal *MSX2A*. We speculate that the activities of alternative splicing enhancers may vary in different cells or developmental stages, making it possible to change protein structure or levels in particular tissues, while still preserving other essential functions of a key developmental gene.

## Methods

### QTL mapping

The JAMA × PAXB cross contains approximately 2600 F2 fish in total, all derived from a single set of outbred grandparents. Multiple pairs of F1s were used to generate different families of F2 progeny [26]. For initial QTL mapping of dorsal and anal spine lengths, we used a set of 375 full-sibling F2s (family 4) that were genotyped at 243 microsatellite markers. Phasing and linkage map construction were performed using tmap [53].

The spine lengths used for mapping were calculated as residuals from a linear model including terms for standard length (DS1 and DS2) or standard length and sex (DS3 and AS). Standard length is defined as the distance from the tip of the upper jaw to the end of the last vertebra (body length excluding caudal fin). QTL scans were performed in R/qtl [54, 55] using Haley–Knott regression. Significance thresholds for single-QTL scans were determined by running 10,000 permutations and using *α*=0.05. Multi-QTL models were identified by using the stepwise function to test additive models iteratively with up to ten QTLs, with penalties determined based on 10,000 scantwo permutations.

For fine mapping on chromosome 4, additional fish were analyzed from F2 families 3, 7, 8, 12, 15, and 23, for a total of 2,002 F2s. The additional fish were genotyped using six microsatellite markers on chromosome 4 (Stn42, Stn45, Stn183, Stn266, Stn292, and Stn309) to identify recombinant animals within a 16.8 cM region. For recombinant animals, morphological measurements of DS1, DS2, and standard body length were taken as described [25]. Additional genotyping was performed for the recombinants using a set of 48 densely spaced markers (37 unique genetic positions; listed in Additional file 1: Table S1). Genotype values were converted to simple marine or freshwater labels, depending on the grandparent of origin, so that different families could be combined and the data could be treated like a single F2 cross between inbred lines. The linkage map was built in R/qtl, and the QTL mapping procedure was similar to the initial family 4 analysis. See Additional file 5 for a full description of both the initial QTL mapping and fine mapping.

### Hybrid generation and tissue dissection

Three marine female sticklebacks from Rabbit Slough, Alaska (RABS), were crossed with a PAXB male stickleback by in vitro fertilization to generate F1 hybrid fish. The fish were raised in a 29-gallon tank with 3.5 ppm Instant Ocean. They were grown to 13 mm standard length and individually sacrificed in an overdose of buffered MS-222. Each fish was immediately dissected to remove the following tissues: first dorsal spine, second dorsal spine, pelvic spines, pectoral fins, caudal fin, dorsal fin, anal fin, cloaca, eyes, upper jaw (premaxilla with oral teeth), lower jaw (approximately the articular and dentary with oral teeth), left anterior flank skin, left posterior flank skin, whole brain, and kidney. As each tissue was removed, it was placed into an ice-chilled 1.5 mL centrifuge tube containing 500 *μ*L TRIzol Reagent (Thermo Scientific, Waltham, MA). After dissection, samples were immediately frozen at −20 °C and transferred to −70 °C within 2 days of the initial dissection.

RNA was then extracted according to the TRIzol Reagent protocol with the following modifications: samples were thawed at room temperature for 5 min, then vortexed for 2 min, and chloroform was used instead of 1–bromo–3–chloropropane. Pellets were resuspended in 20 *μ*L of nuclease-free water (Thermo Scientific), heated at 65 °C for 5 min to resuspend, then treated with RNase-free DNase I according to the manufacturer’s protocol (Thermo Scientific). Because the tissue samples were small, 6 *μ*L of the DNase-treated RNA was used for cDNA synthesis using Superscript III Supermix (Thermo Scientific) according to the manufacturer’s recommendations for random hexamers in a 20 *μ*L reaction. For some samples, an *MSX2A* gene-specific primer was used, but results did not differ from those with random hexamers.

### Allele-specific expression measurements

To produce amplicons that could be used for allele-specific expression tests, PCR primers were designed such that the resulting products were less than 400 bp and included at least one single-nucleotide polymorphism (SNP) that could be used to distinguish the RABS and PAXB alleles. Amplification primers were also required to flank an exon boundary to avoid generating products from residual genomic DNA. Primers for amplification and sequencing were designed by EpigenDx, Inc. (Hopkinton, MA). The forward PCR primer was 5 ^′^-biotinylated and purified by high-performance liquid chromatography (Integrated DNA Technologies, Coralville, IA). PCR amplification from the tissue-specific larval cDNA samples was performed using Phusion polymerase (Thermo Scientific) in at least a 40 *μ*L reaction volume, according to the manufacturer’s recommendations. Touchdown PCR profiles were used to avoid mispriming events on similar sequences. Products were checked for the correct size on an agarose gel, then sent to EpigenDx for pyrosequencing. Control plasmids were generated from the amplified cDNA products for each allele using unmodified versions of the PCR amplification primers. PCR products were TOPO-TA-cloned into the pCR4-TOPO vector (Thermo Scientific), and products were screened so that control plasmids for each gene had the same orientation. Plasmids were purified using the QIAprep Spin Miniprep Kit (Qiagen, Valencia, CA) and quantified in triplicate using a NanoDrop ND-1000 spectrophotometer (Thermo Scientific). Control plasmids were diluted to 100 pg/ *μ*L, 1 pg/ *μ*L, or 10 fg/ *μ*L in 2:1, 1:1, and 1:2 ratios and included along with the PCR reactions for cDNAs.

For *MSX2A*, the following PCR primers were used:

- 5 ^′^-biotin-GCCGGTTTTGGAACCAGATC-3 ^′^ (labeled forward primer, P1)
- 5 ^′^-GTATCCGGCCCCGTTAAGGT-3 ^′^ (reverse primer, P2)
- 5 ^′^-GTTCTCCTCCTCTCTGTT-3 ^′^ (sequencing primer)

The PCR conditions were as follows: denature at 98 °C for 10 sec, anneal at 60 °C for 15 sec, and extend at 72 °C for 20 sec, for 40 cycles. The initial annealing temperature was 70 °C and was reduced by 1 °C/cycle for the first ten cycles. Nucleotide 420 of the coding sequence was analyzed (TGTTGCTGTC[T/C]GAGACCCAGG).

For *CPEB4A*, the following primers were used:

- 5 ^′^-biotin-ATGCATTCCTGCTGTTTCAA- ^′^ (labeled forward primer)
- 5 ^′^-ATACCCTTTGGATACTTGAGTTCA- ^′^ (reverse primer)
- 5 ^′^-GACCCCTCCTACAAAGA- ^′^ (sequencing primer)

The PCR conditions were as follows: denature at 98 °C for 15 sec, anneal at 63 °C for 10 sec, and extend at 72 °C for 20 sec, for 35 cycles. The initial annealing temperature was 72 °C and was reduced by 1 °C/cycle for the first nine cycles. A nucleotide in the antepenultimate exon was examined (CCAGGAAAAC[T/C]ATCTTTGTAG). For both *MSX2A* and *CPEB4A*, a 30-sec 98 °C initial denaturation step and a 7-min 72 °C final extension step were included in the PCR programs. For both genes, a C at the polymorphic site corresponded to the marine allele (RABS) and a T corresponded to the freshwater allele (PAXB).

Pyrosequencing results were reported by EpigenDx in terms of percentages of each nucleotide observed at the SNP site. For *MSX2A*, reactions were performed in triplicate and the results were averaged before analysis. The resulting percentages were arcsine transformed. A two-sided *t*-test with unequal variance (Welch’s *t*-test) was used to compare each tissue group to the 1:1 controls. Average values and standard errors of the mean (SEM) were then back-transformed, and were adjusted for any PCR bias by performing a cubic regression of the pyrosequencing nucleotide percentages against the known input ratios of the control plasmids. See Additional file 6 for details of the calculations. The average ratios of the freshwater allele to the marine allele are presented in Fig. 2.

### Egg microinjection and stickleback transgenesis

Single-cell embryos were injected using custom-pulled glass needles made from microcapillary tubes (World Precision Instruments, no. 1B100F-4) pulled on a Sutter P-97 micropipette puller using the following settings: heat 270, pull 150, velocity 100, time 150, and pressure 500, with a 3.0 mm trough filament. Because the tough chorion of the stickleback egg is resistant to injection, a metal saw blade (Hilti, no. 00374342) was used to hold eggs in place atop a glass plate (from a 5×7-inch frame backing kit) on a microscope stage (Leica S8 APO microscope with S series transmitted light sub-base), with water applied to the eggs to prevent dehydration. Prior to loading onto the stage, fertilized eggs were kept in a small petri dish without water. Paint brushes were used to manipulate eggs gently. A Narishige M-152 manipulator and ASI MPPI-2 pressure injector with micropipette holder and foot switch were used to perform the injections.

Transgenic construct A was derived from the plasmid p817-mgammaFcry-EGFP [56], which contains *eGFP* under the control of the mouse gamma F-crystallin promoter to provide fluorescent labeling in the lens. Clone CH213-38J23 from the CHORI-213 bacterial artificial chromosome library of the Salmon River marine population (mouth of Fraser River, British Columbia) was digested with *XhoI*. A 5.6-kb fragment containing *MSX2A* and surrounding genomic context was cloned into the *XhoI* site of p817-mgammaFcry-EGFP. The vector backbone contains *I-SceI* recognition sites, so the plasmid was co-injected with *I-SceI* meganuclease to integrate the entire expression cassette into the genome [57, 58].

Construct B was derived from our pT2HE construct for enhancer activity tests, which was in turn derived from the Nonet Lab bleeding heart plasmid vector [59] by removing the heart-expressed mCherry marker. The *eGFP* gene was replaced with an *eGFP*-2A-*MSX2A* coding unit to provide co-expression of *MSX2A* and the fluorescent reporter, and five copies of *MSX2A-CNE* were cloned into the *SfiI* site upstream of the *hsp70* promoter. The vector backbone contains *Tol2* inverted repeats, so the plasmid was co-injected with *Tol2* transposase mRNA to promote efficient integration [60–62].

The reporter construct used to visualize the expression pattern of *MSX2A-CNE* contained the same 5x concatemer of the enhancer sequence, which was cloned into the *SfiI* site of pT2HE, upstream of the *hsp70* promoter and *eGFP* gene (Additional file 2: Figure S2). This plasmid was also co-injected with the *Tol2* transposase transcript as described [61]. Full sequences of the plasmids used for transgenesis are provided in Additional file 3.

GFP expression was visualized in embryos using a Leica MZ FLIII microscope with a mercury vapor lamp and GFP Plus filter set (excitation 460–500 nm, emission 510 nm longpass). Fluorescence in adult fish was observed using blue LED light sources with barrier filter glasses and camera filters from NightSea (excitation 440–460 nm, emission 500 nm longpass).

### Outlier analysis

Some individual fish in transgenic experiments 1 and 3 show phenotypic values that lie more than 1.5 times the interquartile range beyond the first or third quartile of the data, classifying them as outliers. Excluding these individuals as outliers does not substantially change the overall conclusions of the transgenic experiments:

Experiment 1: With outliers excluded, the transgenic difference in mean spine length is 0.23 mm (*t*(7.7)=2.32, *p*=0.05, Welch’s *t*-test).

Experiment 3: With outliers excluded, the transgenic difference in mean spine length is 0.20 mm (*t*(16.4)=3.23, *p*=0.005, Welch’s *t*-test).

In addition, in experiment 2, two tanks of uninjected fish grew to larger sizes because of their low rearing density. If these tanks are excluded (both *n*=7) and the tank of GFP-positive transgenic fish (*n*=14) is compared only to the tanks of injected but non-GFP-fluorescent siblings (both *n*=16), the mean transgenic effect would be 0.17 mm (*t*(25)=2.07, *p*=0.05, Welch’s *t*-test).

### RNA in situ hybridization

Probes for in situ hybridization were generated by RT-PCR from poly(A)‘ RNA purified with RNAwiz (Ambion, Austin, TX) from four lab-raised stage 31 larvae [63] from the San Joaquin River in Friant, CA, USA.

The following primers were used to amplify a 765-bp fragment from exon 2, 305 bp of which are in the 3 ^′^ untranslated region:

- 5 ^′^-CCAGGCCAAAAGGTCATTCTC-3 ^′^
- 5 ^′^-GAGGAAGTTTCACCAGAAGC-3 ^′^

This fragment was cloned into the pCR4-TOPO vector (Thermo Scientific), cut with *NotI* enzyme, and transcribed with T3 polymerase (Promega, Madison, WI) as described [9]. Stage 30/31 larvae (20 days post hatching) from a lab-raised in vitro fertilized cross of Matadero Creek fish (Palo Alto, CA, USA) were used for in situ hybridization. RNA in situ hybridization was performed as described [64] with the following modifications: larvae were bleached in a 4:1 mix of 30 % H_2_O_2_ and phosphate-buffered saline with 0.1 % Triton X-100 for 1 hour under bright light. The proteinase K treatment lasted 5 minutes, the hybridization temperature was 65 °C, and the coloration reaction used BM Purple (Roche Diagnostics, Indianapolis, IN).

### Analysis of splicing variants

The following primers were used to generate a PCR amplicon from cDNA, spanning from the *MSX2A* start codon to the stop codon position of the full-length transcript:

- 5 ^′^-ATGTCCTCCGCCGGAGACCC-3 ^′^ (P3)
- 5 ^′^-TCATAAAGCCTGTGGTCCACCTACGG-3 ^′^ (P4)

PCR reactions were performed using Phusion polymerase (Thermo Scientific) with initial denaturation at 98 °C for 30 sec, followed by 35 cycles of the following: denature at 98 °C for 10 sec, anneal at 63 °C for 30 sec, and extend at 72 °C for 25 sec. A 5-minute final extension at 72 °C was included.

Gel bands were extracted using a QIAquick Gel Extraction Kit (Qiagen). Clones of PCR products were obtained using the Zero Blunt TOPO PCR cloning kit (Thermo Scientific). Digests of PCR products were performed with FastDigest *BspCNI* (Thermo Scientific).

## Additional files

Additional file 1 **Table S1**. Markers used for fine mapping on chromosome 4. (XLSX 10 kb)

Additional file 2 Supplementary Figures. **Figure S1.** Allele-specific expression results for *CPEB4A*. **Figure S2.** Reporter construct used to visualize the expression pattern of the *MSX2A-CNE* enhancer. **Figure S3.** *MSX2A* and the *MSX2A-CNE* enhancer sequence. **Figure S4.** Embryonic expression pattern of *MSX2A-CNE*. **Figure S5.** Preferential production of the alternative splice form by the freshwater *MSX2A* allele. (PDF 1135 kb)

Additional file 3 Sequence information. Exon sequencing results, alignments of MSX2A protein sequences, and sequences of plasmids used for transgenesis. (ZIP 42 kb)

Additional file 4 Transgenic analysis. Dorsal spine measurements of PAXB sticklebacks carrying different *MSX2A* transgenes. (ZIP 22 kb)

Additional file 5 QTL mapping data and scripts. Genotype and phenotype data used for mapping spine length QTL in the JAMA × PAXB cross. (ZIP 5024 kb)

Additional file 6 Allele-specific expression measurements. Pyrosequencing data used for calculating *MSX2A* and *CPEB4A* allele-specific expression ratios. (XLSX 36 kb)

## Abbreviations

AS: Anal spine
C: Cloaca
CF: Caudal fin
cM: Centimorgan
CNE: Conserved non-coding element
CPEB4A: Cytoplasmic polyadenylation element binding protein 4A
CSS: Cluster separation score
DS: Dorsal spine
EDA: Ectodysplasin
eGFP: Enhanced green fluorescent protein
F1, F2: First and second filial generations of a cross
GFP: Green fluorescent protein
hnRNP: Heterogeneous nuclear ribonucleoprotein
hsp70: Heat shock protein 70
JAMA: Japanese marine population
LJ: Lower jaw
LOD score: Logarithm (base 10) of odds ratio for QTL model at a given locus
MSX2A: Msh homeobox 2A (muscle segment homeobox)
NKX2-5: NK2 homeobox 5
PAXB: Paxton Lake benthic population (British Columbia)
PCR: Polymerase chain reaction
PF: Pectoral fin
PITX1: Paired-like homeodomain 1: pituitary homeobox 1
PM: Premaxilla
PS: Pelvic spine
pT2HE: Tol2 hsp70 eGFP plasmid vector
PVE: Percentage variance explained
QTL: Quantitative trait locus
RABS: Rabbit Slough marine population (Alaska)
RT-PCR: Reverse-transcription polymerase chain reaction
SEM: Standard error of the mean
SNP: Single-nucleotide polymorphism
STC2A: Stanniocalcin 2A
