# Supplementary material for: Dorsal spine evolution in threespine sticklebacks via a splicing change in MSX2A
Source: BMC Biol. 2017 Dec 7;15:115. doi: 10.1186/s12915-017-0456-5 (PMC5719529; doi:10.1186/s12915-017-0456-5)
Supplement: Supplementary file 2 — Supplementary Figures. Figure S1. Allele-specific expression results for CPEB4A. Figure S2. Reporter construct used to visualize the expression pattern of the MSX2A-CNE enhancer. Figure S3. MSX2A and the MSX2A-CNE enhancer sequence. Figure S4. Embryonic expression pattern of MSX2A-CNE. Figure S5. Preferential production of the alternative splice form by the freshwater MSX2A allele. (PDF 1135 kb) [file 12915_2017_456_MOESM2_ESM.pdf]

Supplementary Figures

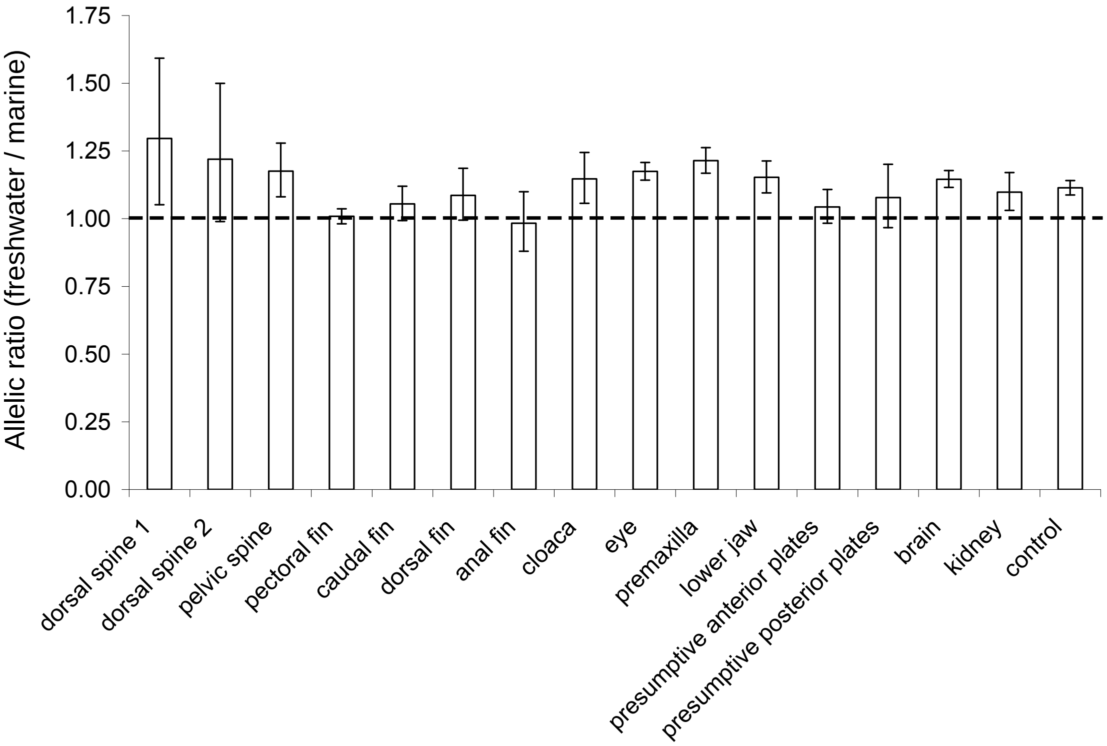

Figure S1: **Allele-specific expression results for *CPEB4A*.** Relative expression of *CPEB4A* alleles in various tissues, calculated as the ratio of freshwater transcripts (PAXB population) to marine transcripts (RABS population). Transcript levels were quantified by pyrosequencing of an intron-spanning PCR product obtained from F1 hybrid tissue samples. No significant differences from the 50:50 plasmid control were found ($p > 0.05$). Error bars represent ratios of standard error of the mean (SEM) for freshwater and marine measurements.

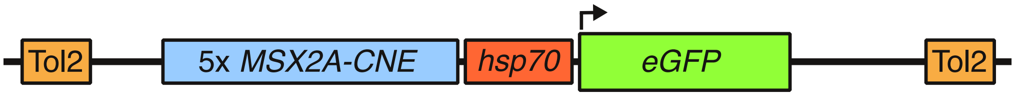

Figure S2: **Reporter construct used to visualize the expression pattern of the *MSX2A-CNE* enhancer.** Five copies of *MSX2A-CNE* were used to drive expression of *hsp70*:*eGFP*, and genomic integration was catalyzed with the help of *Tol2* transposase. The positions of the *Tol2* inverted repeats are shown in orange.

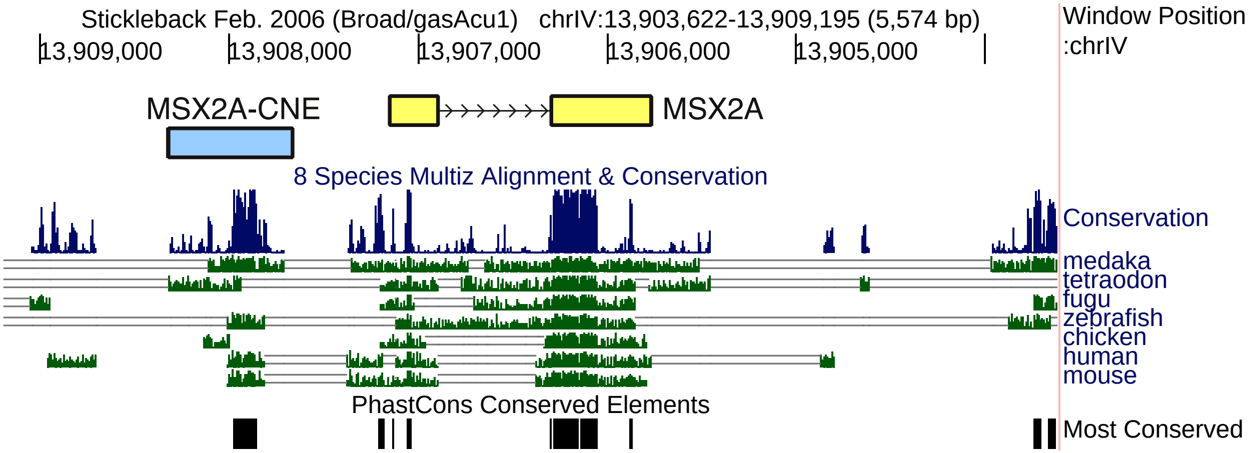

Figure S3: ***MSX2A* and the *MSX2A-CNE* enhancer sequence.** UCSC Genome Browser view of *MSX2A*, with direction reversed to show the gene in 5′ to 3′ orientation. The entire genomic region shown in the window corresponds to the 5.6 kb BAC fragment that was cloned into Construct A for the transgenic rescue experiments. The full-length *MSX2A* coding sequence is shown (yellow), which extends beyond the start and stop predicted in the Ensembl gene model. The position of the *MSX2A-CNE* sequence is shown in blue. An 8-way cross-species conservation track is displayed, showing that *MSX2A-CNE* is partially conserved in mammals.

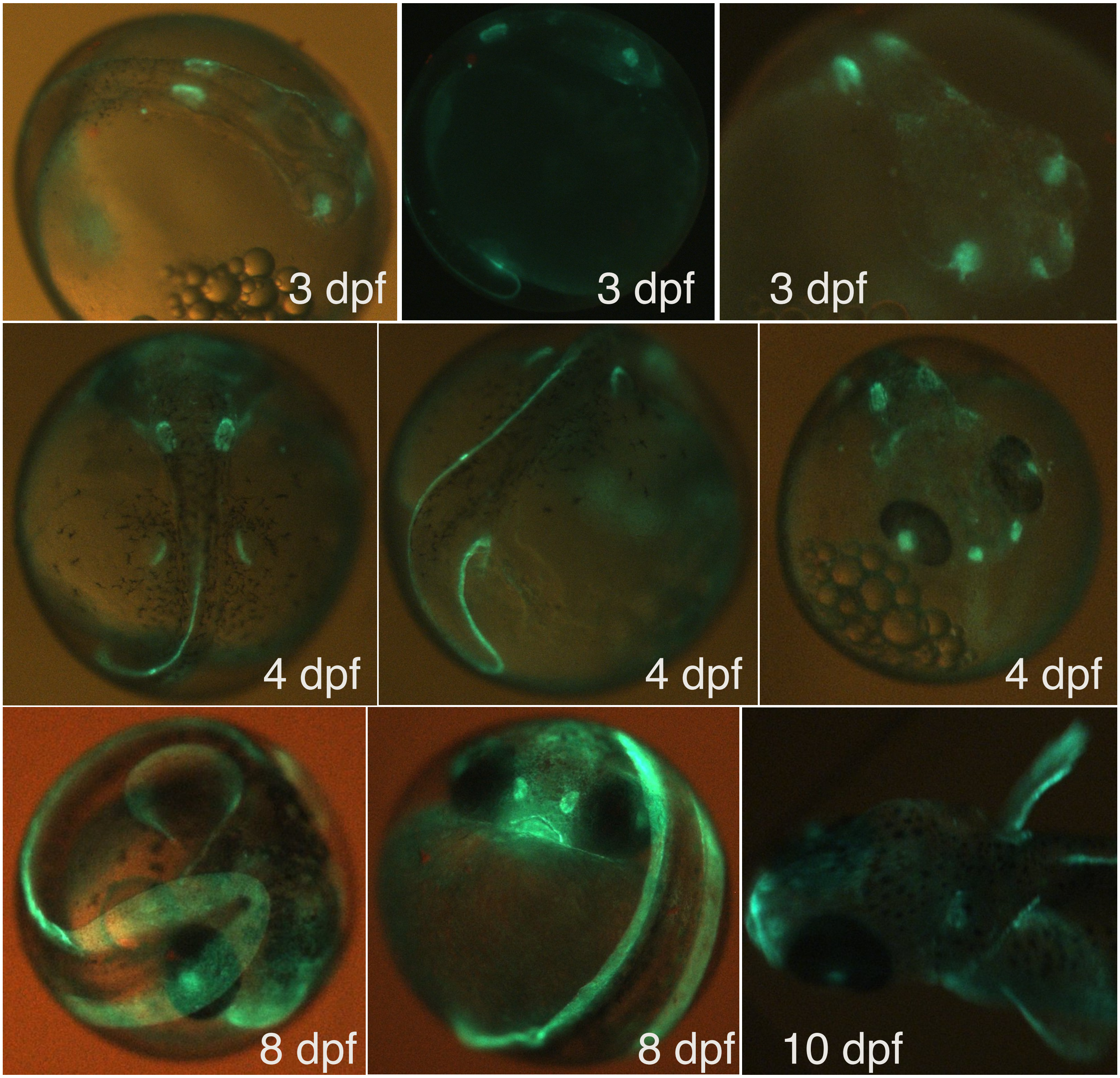

Figure S4: **Embryonic expression pattern of *MSX2A-CNE*.** Expression pattern of *MSX2A-CNE* in a single developing embryo with low mosaicism, from 3 to 10 days post fertilization (dpf). Prominent sites of GFP expression include median fin fold, pectoral fins, the otic and olfactory placodes, and the skin around the mouth. Lens expression can occur with the *hsp70* promoter alone, but may also be part of the expression pattern of this enhancer (lens placode).

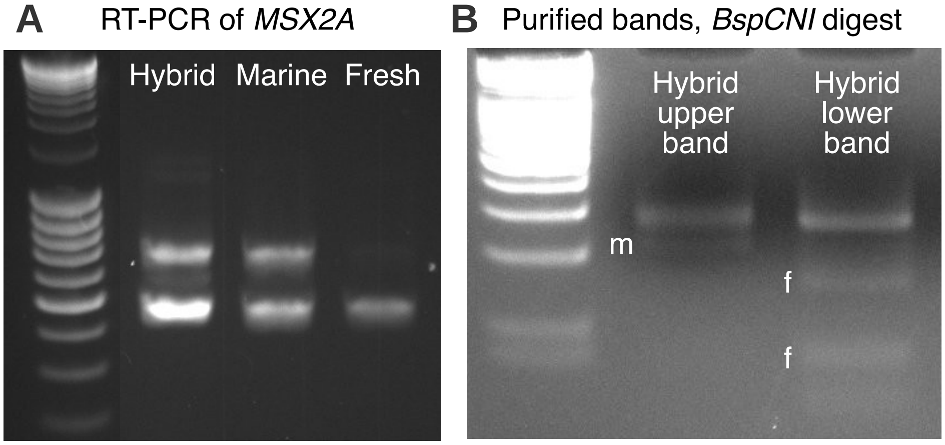

Figure S5: **Preferential production of the alternative splice form by the freshwater *MSX2A* allele.** A) PCR amplification of the *MSX2A* coding sequence from cDNAs prepared from whole stickleback larvae. In addition to the expected full-length sequence (807 bp), an alternative, shorter splice form is observed (584 bp). The short splice form is the predominant form observed in the freshwater sample. B) Extracting the gel bands from the hybrid PCR sample and digesting with *BspCNI* allowed us to characterize the allelic bias within each band. The full-length upper band produces digest products consistent with a marine genotype (m), and the smaller band produces freshwater-specific digest products (f). Image processing: In panel A, additional replicates of the hybrid sample were cropped from the photo. In panel B, brightness and contrast were increased to emphasize bands corresponding to digest products.
